# Ethical and practical issues to consider in the governance of genomic and human research data and data sharing in South Africa: a meeting report

**DOI:** 10.12688/aasopenres.12968.1

**Published:** 2019-05-22

**Authors:** Ciara Staunton, Rachel Adams, Edward S. Dove, Natalie Harriman, Lyn Horn, Melodie Labuschaigne, Nicola Mulder, Antonel Olckers, Anne Pope, Michèle Ramsay, Carmen Swanepoel, Nora Ni Loideain, Jantina De Vries

**Affiliations:** 1 School of Law, Middlesex University, London, UK; 2 Information Law and Policy Centre, Institute of Advanced Legal Studies, University of London, London, UK; 3 Human Sciences Research Council, Cape Town, South Africa; 4 School of Law, University of Edinburgh, Edinburgh, UK; 5 Stellenbosch University, Cape Town, South Africa; 6 Office of Research Integrity, University of Cape Town, Cape Town, South Africa; 7 Department of Jurisprudence, University of South Africa, Pretoria, South Africa; 8 Computational Biology Division, University of Cape Town, Cape Town, South Africa; 9 DNAbiotec (Pty) Ltd, Pretoria, South Africa; 10 Department of Private Law, University of Cape Town, Cape Town, South Africa; 11 Sydney Brenner Institute for Molecular Bioscience, University of the Witwatersrand, Johannesburg, South Africa; 12 Division of Haematological Pathology, Stellenbosch University, Cape Town, South Africa; 13 Department of Pathology, Stellenbosch University, Cape Town, South Africa; 14 National Health Laboratory Services, Tygerberg Hospital, Cape Town, South Africa; 15 Faculty of Medicine and Health Sciences, Stellenbosch University, Cape Town, South Africa; 16 Faculty of Health Sciences, University of Cape Town, Cape Town, South Africa

**Keywords:** data sharing, data protection, governance, ethics, genomics, biobanking

## Abstract

Genomic research and biobanking has undergone exponential growth in Africa and at the heart of this research is the sharing of biospecimens and associated clinical data amongst researchers in Africa and across the world. While this move towards open science is progressing, there has been a strengthening internationally of data protection regulations that seek to safeguard the rights of data subjects while promoting the movement of data for the benefit of research. In line with this global shift, many jurisdictions in Africa are introducing data protection regulations, but there has been limited consideration of the regulation of data sharing for genomic research and biobanking in Africa. South Africa (SA) is one country that has sought to regulate the international sharing of data and has enacted the Protection of Personal Information Act (POPIA) 2013 that will change the governance and regulation of data in SA, including health research data, once it is in force. To identify and discuss challenges and opportunities in the governance of data sharing for genomic and health research data in SA, a two-day meeting was convened in February 2019 in Cape Town, SA with over 30 participants with expertise in law, ethics, genomics and biobanking science, drawn from academia, industry, and government. This report sets out some of the key challenges identified during the workshop and the opportunities and limitations of the current regulatory framework in SA.

## Disclaimer

The views expressed in this article are those of the author(s). Publication in AAS Open Research does not imply endorsement by the AAS.

## Introduction

Genomic research and biobanking have undergone exponential growth in Africa in recent years ( H3Africa Consortium *et al*., 2014). At the heart of this research is the collection and sharing of biospecimens and associated clinical data. Such practices are to be welcomed, as data sharing can limit issues associated with replication, save resources, engender reproducible science, promote new research on existing data sets, and encourage innovation ( ASSAF, 2019; Mulder *et al*., 2017). Overall it can increase the value of the data, leading to advances in biomedical research and improvements in patient care. While this move towards open science is ongoing, there has been a strengthening internationally of data protection regulations ( Dove, 2015), due in part to the coming into force of the EU General Data Protection Regulation (GDPR) in May 2018. These regulations seek to safeguard the rights of data subjects while promoting the movement of data for purposes that include the benefit of research. In this way, they seek to address the tension between open science and the privacy and confidentiality concerns that are inherent in data sharing.

Despite this global shift in the strengthening of data protection regulations, there has been very little consideration of the regulation of data sharing for genomic research and biobanking in the context of low and middle income countries (LMICs), and in Africa, as of 2017, only three countries had enacted regulations on the governance of data sharing for genomic research and biobanking ( de Vries *et al*., 2017). Considering the exploitative nature of research that was pervasive on the continent, the lack of regulations is of concern, as robust national regulations and oversight can guard against it ( de Vries *et al*., 2011; Staunton & Moodley, 2013). Research is for the common good and as such there is an ethical imperative to share data, but it must be non-exploitative, bring reciprocal benefits, promote public trust and minimise social harm ( Yakubu *et al*., 2018).

With this in mind, various policies and guidelines have identified key norms and values that should guide research in resource limited settings. The *San Code of Research Ethics* ( San Council, 2017) focuses on respect, honesty, justice and fairness, care and due process; the TRUST *Global Code of Conduct for Research in Resource Poor Settings* ( TRUST, 2018) puts the values of fairness, respect, care and honesty at the heart of any collaborative research. Specifically for genomic research, Ubuntu, human dignity respect, equity, distributive justice and reciprocity guided the deliberations of the Academy of Science of South Africa (ASSAF) Report on Human Genetics and Genomics in South Africa ( ASSAF, 2019) and the H3Africa *Ethics and Governance Framework for Best Practice in Genomic Research and Biobanking in Africa* ( H3Africa, 2018) is guided by the principles of solidarity or communal-based worldviews, fairness, equity and reciprocity. The values emanating from these policies and guidelines should underpin the development of data protection regulations in Africa, but there is a real risk that institutions in Africa currently lack consistent and coherent policies and standards to govern data sharing.

South Africa (SA) is one country in Africa that has sought to regulate the international sharing of data and has enacted the Protection of Personal Information Act (POPIA) 2013. Although not yet in force, it will change the governance and regulation of data in SA, including health research data. Whilst it is intended that Codes of Conduct are to be developed to guide the implementation of the POPIA, for the higher education sector in SA, it has become increasingly obvious that the governance of data sharing is a concern for researchers in SA as they continue to build upon their collaborations in Africa and around the world.

To identify and discuss challenges and opportunities in the governance of data sharing for genomic and health research data in SA, a two-day meeting was convened in February 2019 in Cape Town, SA. Over 30 participants with expertise in law, ethics, genomics and biobanking science were drawn from academia, industry, and government, primarily from SA and also from the continent more broadly. The workshop discussed a number of significant challenges relating to the governance of data sharing of genomic and human research data in SA, and Africa more broadly, and identified a number of actionable next steps. It is clear that further research is required to address the issue comprehensively. This report sets out some of the key challenges identified during the workshop, the opportunities and limitations of the current regulatory framework in SA.

## Key challenges

It is clear that the sharing of human research data in Africa is faced with considerable legal, ethical, social and technical challenges ( Mulder *et al*., 2017). The technological challenges highlighted include transferring large datasets, particularly to the African region. Workshop participants were informed about a large dataset that took 90 days to be transferred to an Africa-based research institution from the USA and that the process of un-encryption and re-encryption can take a week alone for large, complex data. The costs of data storage, processing and analysis can be considerable and there is a need for training in data capture, transfer, storage and analysis. The focus of the workshop was however on challenges in the governance of data sharing in Africa.

### Broad consent

Discussions at the workshop made it clear that the acceptability of broad consent for genomic research continues to be subject to debate in Africa ^1^. The experience of many participants highlighted the reluctance of many research ethics committees (RECs) in Africa to approve studies that adopt broad consent. The introduction and use of data access committees as an additional layer of governance is evolving, but it was highlighted that it is currently unclear how these committees are working in practice. While the ethical debate on the use of broad consent continues, broad consent nevertheless is currently adopted for many genomic studies across Africa. Its use is only proper if subject to appropriate oversight and governance procedures that foster trustworthiness by protecting personal data while promoting research that has social value ( de Vries *et al*., 2015; Tindana & de Vries, 2016; Yakubu *et al*., 2018)).

There was considerable debate throughout the workshop as to the legal status of broad consent under POPIA in SA. A general prohibition on the processing of ‘special information’ that includes genetic data is imposed by section 26 of POPIA. Exceptions to this are if the data subject consents, the processing is for research purposes, it is disproportionate to ask for consent, or if the Information Regulator has authorised processing with appropriate safeguards in place. Section 13 of POPIA requires personal information to be collected for a ‘specific, explicitly defined and lawful purpose’ and secondary use of the information beyond that specified in the original consent form is only permitted if it is for research intended to improve health (S.15(3)(d)(i)) and the information will not be published in an identifiable form (S.15(3)(e)). The view was expressed by the representatives of the Office of the Information Regulator that these specific requirements will stop the use of broad consent once POPIA is in force. However many legal academics in attendance also pointed out that a purposive interpretation of POPIA permits broad consent for research in SA, particularly when one considers the provisions of Section 2 it states the purpose of the legislation is to give effect to the constitutional right to privacy by safeguarding personal information, subject to limitations that seek to protect ‘important interests, including the free flow of information within the Republic and across national borders’. Such an interpretation also aligns with the current Department of Health 2015 Ethics in Health Research Guidelines that permits broad consent ( DoH, 2015). Undoubtedly clarity is necessary as to the legal status of broad consent, but a purposive interpretation of POPIA suggests that it is permitted in SA. However, this is not to suggest that it is legally mandated. Rather it is one of a number of consenting models that researchers may adopt and it is for RECs to decide whether it is ethically permissible, or if another consenting model, such as specific or tiered consent, is preferable.

### Community engagement

Under POPIA, the Information Regulator has a public engagement role and is required to consult and engage with the public on matters relating to personal information. This public engagement role is to be welcomed, but it is contingent on the provision of appropriate funding to enable the Information Regulator to fulfil this role.

The workshop also discussed the importance of community engagement (CE) in supporting the implementation of broad consent and research generally. CE is seen as critical in providing for the ethical conduct of research and can help ensure that the community receives reciprocal benefits from research. However, key challenges in the successful implementation of CE were highlighted. The long-standing issues of identifying the ‘community’ was raised as they may not be a distinct or homogeneous group. The focus tends to be on simply informing the community of the purpose or intentions of the researchers, with little effort made to bridge the knowledge gap between researchers and communities. The workshop heard about the experiences of the West African Ebola outbreak where biospecimens were circulated all around the world, creating a ‘virtual biobank’ of West African biospecimens. Through CE, participants insisted that the biospecimens should be returned so that they could be governed by the country of its origin. The workshop ended with a call for community engagement PLUS in Africa, that is Public Learning and Understanding of Science and Social Science. This would involve the community becoming aware of their rights to empower them to negotiate on tangible returns.

### Institutional challenges

The difficulties of centralising and standardising research ethics and compliance with data sharing at a university level was discussed. Major concerns related to the lack of adequate training for researchers on these issues, as well as the lack of clear guidelines from government regarding the specific regulatory requirements of managing health-related data. With regard to compliance with data protection law, it was noted that universities fail to differentiate between its handling of research data and institutional administrative data. The ongoing work of the Universities of South Africa (USAf) in drafting a Code of Conduct for use by universities was highlighted, as under section 60 POPIA, the Information Regulator can authorise a Code of Conduct. However, there was concern expressed that this Code is primarily focusing on institutional administrative data and that issues specific to research data may be neglected or overlooked. There are considerable differences in the legal and ethical requirements in the processing of institutional administrative data and human research data. As such, it was clear that there is a need for a Code of Conduct for researchers on the duties, obligations and safeguards necessary in the use of personal data for research purposes. This is particularly pertinent and needed to help balance the competing interests of the protection of the data subject and the community as academia moves towards open science, with the need to maximise the social value of the data and research.

The traditional independence of academics was also raised as a concern as they often work in silos. Management structures for the use of personal information are often lacking and where they are in existence, there are different management layers for the different types of data. The appointment of Information Officers (IO) under section 55 of POPIA should assist universities in resolving some of these challenges. They are to be appointed by the responsible party and registered with the Information Officer before they can take up their role. The IO is expected to encourage compliance, deal with any data requests, and be involved in any investigation by the Information Regulator. In this way section 55 brings clear lines of accountability and by acting as a conduit, the IO can ensure that research institutions are accountable to the data subject and the Information Regulator. However, there is limited guidance within the POPIA on the IO, the qualifications or experience required, whether the IO can be involved in the processing of personal information or if they can be contracted out. The Information Regulator must develop a job description detailing the duties and responsibilities of the IO as well as a person specification. Failure to do so risks the responsibility of the IO falling to someone currently within the institution that may not have the necessary skills, experience or time. Furthermore, the IO must be adequately resourced (in terms of time, infrastructure, staff and finances) with funding ring fenced to ensure that they can fulfil their duties. Other concerns raised included the role of RECs (discussed below), the expense of the software systems needed to manage and share large datasets, as well as the potential risks to universities of non-compliance including fines, reputational damage, legal disputes and even the loss of large datasets through third party providers.

### Research ethics committees

Under POPIA, the further processing of personal information under section 15(3)(e) is only permitted if the responsible party is satisfied that its use is for research purposes and that the results will not be published in an identifiable form. In the research context, this responsibility for review will most likely be delegated to RECs. Some concerns regarding the ability of RECs in Africa to conduct this review were expressed. First, the oversight of data protection and data sharing requires particular scientific expertise. RECs as currently constituted may not have the adequate expertise and manpower to appropriately review such research protocols and this must be addressed through training and staffing of personnel with adequate expertise. Second, RECs may not have received adequate training on the legal implications of the POPIA and GDPR as it relates to research in SA. Third, it was noted that RECs can act as gatekeepers with an over-cautious approach to research ethics and compliance as they operate within frameworks that are primarily protectionist in nature. There is a critical need to move beyond the privacy and confidentiality paradigm of data processing regulation, and to embed those ethical values and principles that have particular importance for the African region, including equity, reciprocity and solidarity. Finally, it was noted that RECs are currently overworked and under-resourced. Additional oversight and regulatory requirements that are introduced as a result of data protection legislation will likely only serve to increase the burden of RECs who already have a large review burden and likely result in increasing delays in reviews.

There was a call for the development of a national policy on data access. It was argued that this policy should be developed in conjunction with the Department of Science and Technology (DST) and built into the Department of Health’s ethics guidelines. This policy could be disseminated through the National Human Research Ethics Council to RECs and assist researchers and RECs in the management and oversight of data.

### Resource constraints

Constraints regarding resources was a major and cross-cutting concern raised. A lack of adequate resources impacted on compliance levels by research institutions, the capacity of RECs, the extent to which researchers could adequately engage in CE and consent processes, as well as the provision of training to researchers and next generation researchers, or students, on research ethics and data protection compliance. This concern also applied to the Information Regulator, established under section 40 of POPIA. The remit of the Information Regulator is considerable as they are required to provide education, monitor and enforce compliance, consult with interested parties where necessary, handle complaints, conduct research and report to Parliament when necessary, develop codes of conducts and facilitate cross-border cooperation. It will have an essential role in ensuring compliance, accountability and fostering trust in the protection of personal information in SA. To adequately fulfil its role, the Office of the Information Regulator must have the necessary resources to carry out its functions. Its staff should include those with expertise in the management and protection of health data for research and it must be proactive in engaging with those involved in research. Of importance is that the Office of the Information Regulator be granted adequate resources to fulfil its mandate and carry out its enforcement functions, including resources to monitor and investigate.

### Private sector

It was noted that the boundary between the public and private sector is becoming increasingly fluid and going forward, the question is how to align these two groups. The private sector is not a homogeneous group and the interests of multi-nationals *vis-à-vis* small or medium-sized enterprises may differ. What is clear is that there is a need to ensure, as in all other sectors, that the processing of personal information within the private sector is ethical and compliant with POPIA.

A key concern highlighted for industry relates to ownership of data. Relatedly, it was discussed how data sharing agreements between research institutions and the private sector must be transparent and the terms unequivocal, in order to promote accountability and build trust with the public. When the private sector accesses and uses data, there must be accountability to ensure that the data is used appropriately. A suggestion was made that in the negotiation of these transactions and agreements, it would be beneficial to have an independent and experienced negotiator on both sides. It was further discussed how in terms of non-compliance, the private sector responds most effectively to monetary penalties and will change undesirable practices if regulation is clear. In relation to the Code of Conduct for research, it should specifically mention the private sector and include requirements for collaboration with industry partners and commercialisation of research.

### International challenges

The importance of compliance with GDPR if researchers want to access European Union (EU) funding was highlighted. It was also noted that POPIA is less prescriptive than GDPR and so compliance with the provisions of POPIA would not equate to compliance with GDPR. The need for policy toolkits for researchers relating to GDPR and POPIA in terms of human research data specifically were called for.

The key issue regarding GDPR discussed at the workshop pertained to the issue of legal avenues for the international transfer of personal data, such as the provision whereby data can be transferred internationally to a recipient country whose relevant legal framework has been assessed by the European Commission as having an ‘adequate level of protection.’ However, to date, the Commission has recognised only a small handful of countries as adequate, and it was noted with concern that it often takes the European data protection regulators several years to make an adequacy decision about another country’s level of data protection regulation. That being said, it was further discussed how there are other provisions under GDPR providing for data transfer, including the existence of data sharing agreements between organisations in the various countries involved, e.g. contractual clauses between the sender and recipient that are authorised by the competent data protection authority. Also, it was noted that codes of conduct constitute another possible avenue for international data transfers. However, to date, the European Data Protection Board has not approved any code following the process laid down in Article 40 of GDPR.

## Conclusions

Robust governance of genomic and human research data and data sharing is essential for genomic research in Africa. Pertinent challenges include the lack of data protection legislation in Africa, and the tension between the push for open science by funders and many researchers whilst regulators are seeking to protect the security and confidentiality of the data. From this workshop it appears that researchers in SA are currently struggling with issues around data protection, data sharing and risk management and there is a clear need for clarity as to the duties, obligations and responsibilities of all parties involved in collecting, storing and using health research data. It is clear that with the coming into force of POPIA there is a need for transparency and clear lines of accountability to ensure that POPIA is appropriately implemented and that legal compliance is in line with other national guidelines and regulations governing genomic and health research. Lack of clarity may result in a culture of non-compliance that may significantly hinder the opportunities of African-based research institutions to develop cutting-edge research and compete for research funding on a global level.

## Data availability

### Underlying data

No data are associated with this article
