# Supplementary material for: Ethical and practical issues to consider in the governance of genomic and human research data and data sharing in South Africa: a meeting report
Source: AAS Open Res. 2019 May 22;2:15. [Version 1] doi: 10.12688/aasopenres.12968.1 (PMC7118803; doi:10.12688/aasopenres.12968.1)
Supplement: Peer review history

# Peer review: Reviewer response for version 1

**DOI:** 10.21956/aasopenres.14048.r27047

**Published:** 2019-08-05

**Authors:** Obiajulu Nnamuchi

**Affiliations:** 1 Law Faculty, University of Nigeria, Enugu, Nigeria

The paper was well-researched, the language was simple and clear, the main points critically analyzed and the sequencing of arguments quite logical and coherent. Particularly pertinent to my recommendation of the paper for publication is the clarity of the statement of the problem underlying the research – that is, the challenges associated with regulation of data sharing for genomic research and biobanking in Africa as exemplified by South Africa and its recent legislative framework. The authors not only adequately discussed the challenges (broad consent, community engagement, institutional difficulties, research ethics committees, resource constraints, private sector as well as international challenges) but painstakingly showed that the obstacles are not insurmountable by pinpointing the paths to success.

I confirm that I have read this submission and believe that I have an appropriate level of expertise to confirm that it is of an acceptable scientific standard.

# Peer review: Reviewer response for version 1

**DOI:** 10.21956/aasopenres.14048.r27045

**Published:** 2019-07-30

**Authors:** Ellen Wright Clayton

**Affiliations:** 1 Center for Biomedical Ethics and Society, Vanderbilt University Medical Center (VUMC), Nashville, TN, USA

This article reports the proceedings of a two day meeting in South Africa to discuss issues in consent and governance of genomic and human research data. The article is very well written and thoughtful in its discussion, alluding to the issues that are being faced around the world about the need for and barriers to acceptable data sharing and the very complex ethical issues presented by the growing amount of data and the tension between individual and social interests. The article does an especially fine job of discussing the particular challenges in the African context in general as well the uncertainty of the impact of the Protection of Personal Information Act in South Africa, which provides additional control to individuals, albeit not as much as the GDPR. This is a terrific contribution to the international debate.

I confirm that I have read this submission and believe that I have an appropriate level of expertise to confirm that it is of an acceptable scientific standard.

# Peer review: Reviewer response for version 1

**DOI:** 10.21956/aasopenres.14048.r27043

**Published:** 2019-07-16

**Authors:** Rebekah E. McWhirter

**Affiliations:** 1 Centre for Law and Genetics, Faculty of Law, University of Tasmania, Hobart, Tasmania, Australia

This is a timely contribution to the genomic and research data sharing literature. While this topic has been discussed in great depth in relation to the US and European countries, there is relatively little work exploring the governance issues in low and middle income countries. This report of a two-day meeting examining these issues in South Africa (and, to some extent, Africa more broadly) is therefore an important topic and a useful addition.

 It is well-written, with a clear structure, and identifies a number of key unresolved areas ready for future research and policy development. I found the identification of practical challenges particularly valuable, as well as the section highlighting the ways in which different countries in this region focus on different values (especially solidarity, communitarian values, and fairness) in guiding research ethics to those commonly emphasised in the West.

 The workshop was clearly very productive, and the resultant paper is helpful for further understanding the implications of the GDPR at a very practical level.

I confirm that I have read this submission and believe that I have an appropriate level of expertise to confirm that it is of an acceptable scientific standard.

# Peer review: Reviewer response for version 1

**DOI:** 10.21956/aasopenres.14048.r26950

**Published:** 2019-06-21

**Authors:** Simisola O. Akintola

**Affiliations:** 1 Department of Private and Property Law, Faculty Of Law, University of Ibadan (UI), Ibadan, Nigeria

This is a valuable contribution to the literature on ELSI of data sharing and African genomics that fills an important gap. It elaborates practical, ethical and legal perspectives in clear and lucid language. Most contributions in this area do not focus on the legal perspective.

 The paper is a report of a workshop that discussed the significant challenges relating to governance of data sharing of genomic and human research data in South Africa (SA). The paper mentions the key challenges as including technology, consenting models, review and oversight mechanisms of human research data.

 The paper identifies the acceptance of the concept of broad consent as a model for governance in Africa, and it also discusses in detail the provisions of the Protection of Personal Information Act (POPIA) 2013 in this regard. One of the key suggestions of participants on an appropriate consent model is for the model to be based on the norms and values identified at the workshop. This will enable an indigenously accepted model based on the value belief system of the people. However it is noteworthy that there are disagreements on whether broad consent should be permitted or whether tiered consent should be preferred. There are also arguments for dynamic consent in the literature but as discussed in the literature, RECs on the continent are uneasy about broad consent. So the question of the model as suggested would enjoy an enduring solution if based on the values and norms of the people.

 The question of ownership of data is another significant challenge in governance of data sharing that was raised in the report. While it discusses the public and private sector interests in data, and the opinion of participants on it, the position of POPIA on ownership of human research data remains debatable.

 On the whole the workshop and this report fills an important gap in the ongoing discourse on data sharing, and it takes the discussion further by documenting opinions, suggestions and analysis of POPIA from multiple perspectives.

I confirm that I have read this submission and believe that I have an appropriate level of expertise to confirm that it is of an acceptable scientific standard.
